# Sustainable Power Generation in Continuous Flow Microbial Fuel Cell Treating Actual Wastewater: Influence of Biocatalyst Type on Electricity Production

**DOI:** 10.1155/2013/713515

**Published:** 2013-12-25

**Authors:** Zainab Z. Ismail, Ali Jwied Jaeel

**Affiliations:** ^1^Department of Environmental Engineering, Baghdad University, Baghdad, Iraq; ^2^Department of Civil Engineering, Wasit University, Wasit, Iraq

## Abstract

Microbial fuel cells (MFCs) have the potential to simultaneously treat wastewater for reuse and to generate electricity. This study mainly considers the performance of an upflow dual-chambered MFC continuously fueled with actual domestic wastewater and alternatively biocatalyzed with aerobic activated sludge and strain of *Bacillus Subtilis*. The behavior of MFCs during initial biofilm growth and characterization of anodic biofilm were studied. After 45 days of continuous operation, the biofilms on the anodic electrode were well developed. The performance of MFCs was mainly evaluated in terms of COD reductions and electrical power output. Results revealed that the COD removal efficiency was 84% and 90% and the stabilized power outputs were clearly observed achieving a maximum value of 120 and 270 mW/m^2^ obtained for MFCs inoculated with mixed cultures and *Bacillus Subtilis* strain, respectively.

## 1. Introduction

In recent years, interest in microbial fuel cells (MFCs) has grown considerably not only because they provide a way to generate electricity but also because they can be coupled with wastewater treatment [1]. Microbial fuel cells (MFCs) are bioelectrochemical systems that generate electricity by oxidation of organic or inorganic substrates catalyzed by microorganisms [2]. The electrons generated from the oxidation of organic substrates by microbes are generally transferred to a high potential electron acceptor such as dissolved oxygen in the medium. In MFCs, electrons are transported to an insoluble electrode (anode) through an electrical circuit to reach the cathode, where electron acceptors are reduced. As the current then flows over a resistance, electrical energy is directly generated from the MFC [3]. Some researchers have reported the generation of electricity using sludge as the source of microorganisms [4]. However, when using a mixed community, the electrochemical activity of a few bacterial species enhances the power output of the whole system [5, 6]. Hence, it becomes difficult to ascertain the mechanisms and roles of the individual microorganisms contributing to power generation. Pure and mixed cultures of organisms are used to inoculate MFCs but due to high costs, pure microorganisms may not be suitable for the practical operation such as treatment of industrial effluents. Mixed cultures (i.e., soil and wastewater) containing significant amounts of electrogenic bacteria can be used as the cost-effective inoculate for MFCs. However, the nonelectrogenic bacteria (i.e., methanogenic bacteria and denitrifying bacteria) in mixed cultures consume organic substrates without generating electricity [7]. Recently, a number of bacteria such as *Shewanella putrefactions*, family of *Geobacteraceae*, *Rhodoferax ferrireducens*, *Bacillus subtilis*, *Geobacter sulfurreducens*, and *Escherichia coli *were reported in the literature which have ability to transfer produced electrons from oxidized fuel (substrate) to the electrode without using artificial mediator, making it possible to establish mediator—less MFC [8–13]. Although some factors, such as dissolved oxygen, pH, proton exchange material, and cathode were examined well to improve the performance of MFC, the biofactors' effects are not yet well studied, in particular the comparison between single and mixed culture.

In this paper an upflow dual-chamber MFC system was constructed to compare the bio-factors, mainly inoculums species affecting bioelectricity production. The objective of this work is to better understand the effects of the inoculum type and to optimize the performance of MFC fed with primarily clarified actual wastewater.

## 2. Materials and Methods

### 2.1. Microorganisms and Cultures Conditions

In this study, two types of inoculum were tested, *Bacillus subtilis* and activated sludge. The pure culture (*Bacillus subtilis*) was isolated from the mixed cultures (activated sludge). Before inoculating the anodic chamber of the MFC with the isolated culture (*Bacillus subtilis*), the *Bacillus* cells were grown in 25 mL of sterilized M9 medium with 0.2% glucose in a 250 mL Erlenmeyer flask on an orbital shaker (200 rpm) in order to enrich the cultures seeds. The composition of the M9 media used for cultures enrichment was given by Nimje et al. [11] as the mineral salts media (MSM) composed of 1 g/L NH_4_Cl, 3 g/L KH_2_PO_4_, 6 g/L Na_2_HPO_4_, 5 g/L NaCl, 1 mmol/L MgSO_4_, and 0.1 mmol/L CaCl_2_. Prior to sterilization of the prepared MSM, the pH of the media was adjusted to 7.0 with NaOH. The media were sterilized at 121°C for 20 min without glucose which was added afterwards. Glucose was used as the initial electron donor for the anode media. All experiments were carried out at 30 ± 2°C. Mixed cultures (activated sludge) were freshly collected from a local sewage treatment plant (Baghdad, Iraq). The activated sludge was filtered through 0.25 mm pore size filter to remove large particles before inoculation.

### 2.2. MFC System

**Figure 1:**
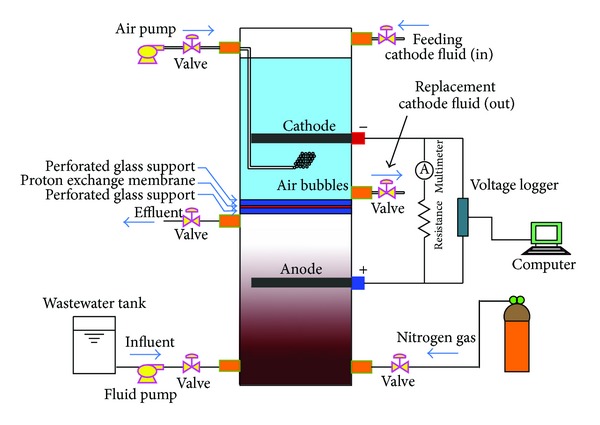
Schematic diagram of the MFC.

The upflow MFC consisted of a two rectangular chambers made of transparent acrylic parallelepiped having dimensions of 52 × 9.4 × 9.4 cm. The cathode chamber (26 cm height) was located on the top of the anode chamber (26 cm height) as illustrated in Figure 1.

**Figure 2:**
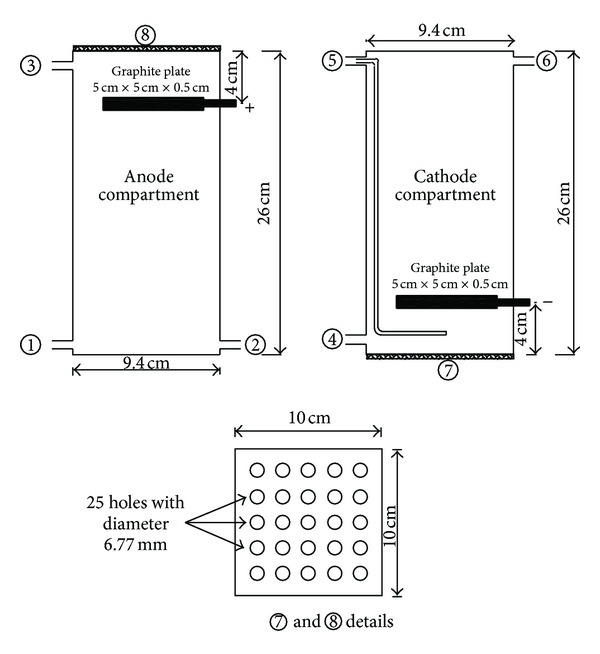
Dimensions of anode and cathode chambers; (1) influent inlet, (2) nitrogen gas inlet, (3) effluent outlet, (4) cathode fluid outlet, (5) air inlet, (6) cathode fluid inlet.

Graphite plain electrodes were used in the MFC; each had a surface area of 60 cm^2^. The graphite electrodes were abraded by sand paper to enhance bacterial attachment. The two chambers were separated by a cation exchange membrane (CEM) type CMI-7000, supplied by membrane international INC., NJ. The CEM sheet of dimensions 10 cm × 10 cm was placed between two perforated glass sheets containing 25 pores, each of 6.77 mm diameters (Figure 2).

Before establishing the construction and setup of the MFCs systems, all the components of the microbial fuel cells were cleaned very well with proper detergent and significantly and repeatedly rinsed with tap water and then with distilled water. The membrane was treated with sodium chloride solution for 6 h and then rinsed with deionized water to ensure good conductivity for protons. Upon constructing and assembling of the MFC, both the anodic and cathodic compartments were filled with deionized (DI) water, gently shaken, and then emptied followed by tight closing of all ports. The anode, in particular, was pretreated and sterilized with boiled distilled water for 1 h and then washed and retreated for additional 30 min using fresh boiled distilled water to insure the sterilization process. Two identical MFCs were used in this study, one of them inoculated with activated sludge and the other inoculated with single culture (*Bacillus subtilis*). The cathode compartment for each MFC was filled with phosphate buffer solution (PBS) as the catholyte solution. This solution consisted of 20.74 g/L Na_2_HPO_4_, 3.11 g/L NaH_2_PO_4_, and 32.93 g/L K_3_Fe(CN)_6_. The bioreactors were operated at temperature 28 ± 2°C and continuously fed with actual wastewater at a rate of 0.1 mL/min until obtaining stable power output. The average initial COD concentration in wastewater was 350 mg/L. Wastewater fed to the bioelectroreactor had a pH ranging from 7.1 to 7.4. The freshly collected wastewater was obtained from the main sewer pipe (Al-Kut city, Iraq).

### 2.3. Analytical Methods

The concentrations of chemical oxygen demand (COD) were determined according to the procedures outlined in the *Standard Methods* [14]. Voltage was continuously measured by a multimeter with a data acquisition system and converted to power according to *P* = *I*∗*V*, where *P* is the power, *I* is the current, and *V* is the voltage. The power was normalized by the surface area of the anodes. Columbic efficiency was calculated as the total coulombs measured divided by the moles of COD removed assuming 4 moles of electrons/mole of COD.

## 3. Results and Discussion

### 3.1. Effect of Inoculums Species on COD Removal

**Figure 3:**
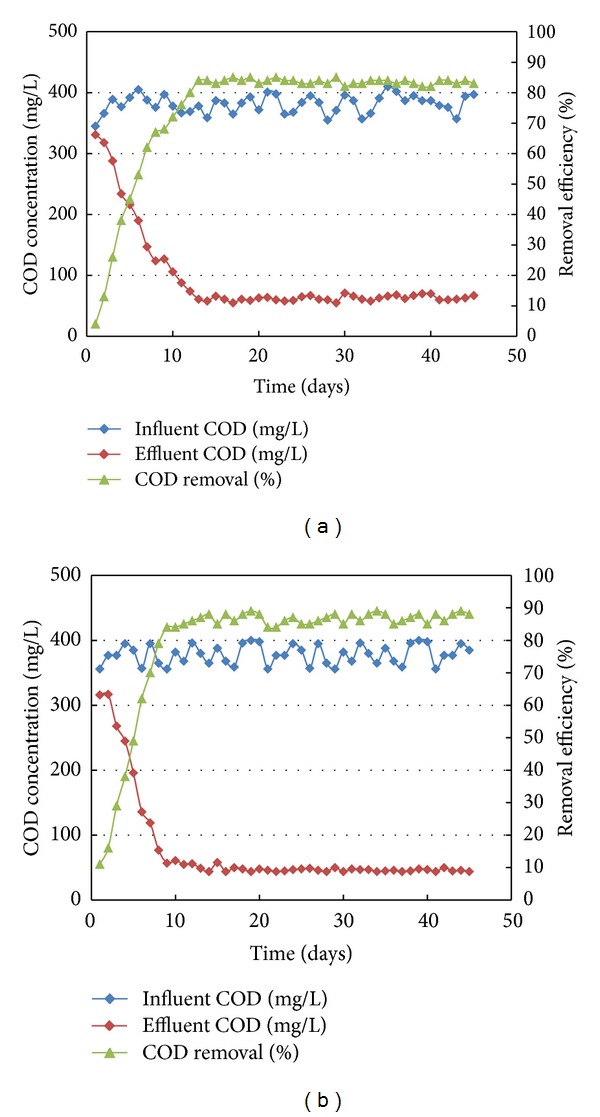
Profile of COD removal in MFC inoculated with (a) mixed culture (b) single culture.

*Bacillus subtilis* and activated sludge were, respectively, inoculated to the anode chambers in two identical MFCs. For each system, the inoculums were maintained without wastewater feeding for 7 days till biofilms attached to the anode surfaces were well observed. Thereafter, the actual wastewater was fed to the anode chamber with COD initial concentration of 350 mg/L to support the formation of biomass and subsequent adaptation to the new microenvironment. Constant COD removal and voltage output were considered as indicators to assess the stable performance of the MFCs. Microbial fuel cells were operated continuously for more than 45 day. Approximately, after 12 days of continuous operation, a steady-state condition was achieved. Maximum COD removal up to 84% and 90% was obtained for MFC inoculated with activated sludge and *Bacillus subtilis* as given in Figures 3(a) and 3(b), respectively.

**Figure 4:**
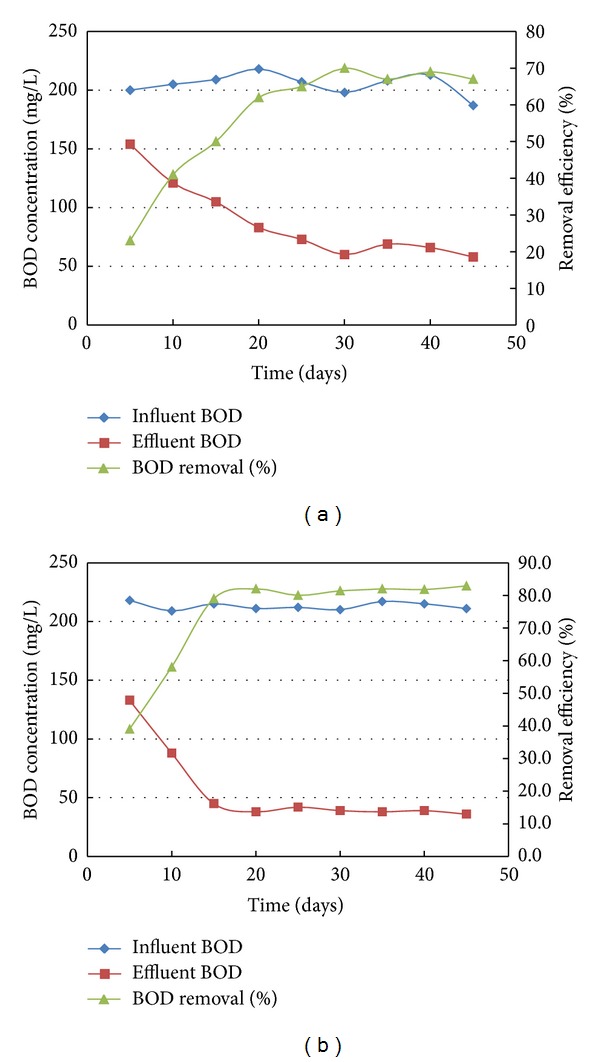
Profile of BOD removal in MFC inoculated with (a) mixed culture (b) single culture.

Although the performance of microbial fuel cells was mainly evaluated in terms of chemical oxygen demand (COD), measurements of biological oxygen demand (BOD) were also carried out for the MFCs. As shown in Figures 4(a) and 4(b), BOD reduction was 70% and 82% for MFCs inoculated with activated sludge and *Bacillus subtilis*, respectively. These results indicate that the rate of oxidation of substrates in terms of COD and BOD by microbes was higher in MFC inoculated with *Bacillus subtilis *compared with that inoculated with mixed cultures.

### 3.2. Power Generation

**Figure 5:**
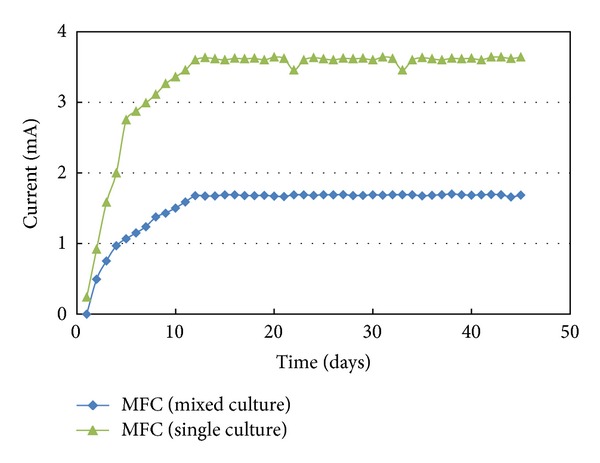
Variation of the generated current with time.

To compare the electricity production and the effect of external resistance between the activated sludge and the pure culture *Bacillus subtilis* on the performance of the MFC, the polarization curves were made by changing the external resistance as function of current to obtain the cell voltage and consequently determine the power output when MFCs were operating at steady state. As shown in Figure 5, the current increased rapidly for the first 12 days to maximum constant values of 1.67 and 3.60 mA for MFCs inoculated with activated sludge and single culture, respectively.

This variation could be attributed to the fact that some types of microbes in the mixed culture which may not be electrochemically active species could compete with active microorganisms including *Bacillus subtilis* for the available substrate and limit their electrochemical activity. The current was maintained stable for 45 days under the given conditions. The open circuit potential was 0.78 volt and the maximum closed circuit voltage drop across a continuous external resistance 250 Ω was 0.42 volt for MFC inoculated with activated sludge.

**Figure 6:**
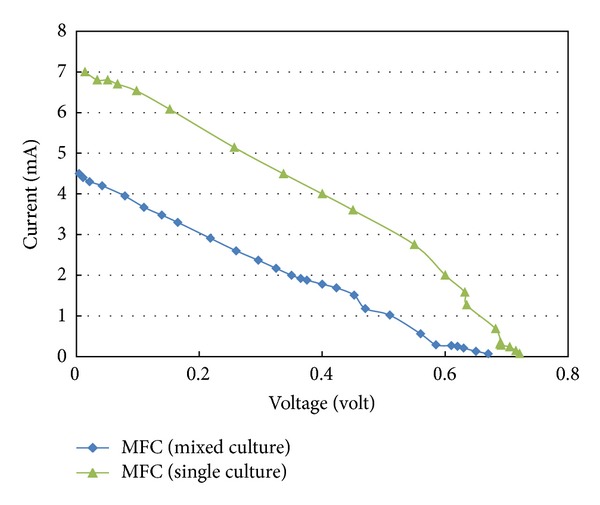
Voltage-current relationship at different external resistances.

For MFC inoculated with *Bacillus subtilis,* the open circuit potential was 0.81 volt and the maximum closed circuit voltage drop a cross continuous external resistance 125 Ω was 0.45 volt (Figure 6). As reported by Nimje et al. [11], *Bacillus subtilis* is one of the most commonly used hosts in fermentation production, because it is simple to cultivate and its products, the protein and metabolites, are often secreted in the growth medium. Also, the composition of M9 medium supplemented with glucose was employed to culture bacteria in the anode with the possibility that biofilm might cause maximum productivity. However, the findings of this study with respect to power generation are not in agreement with the previously reported data relative to the differences of inoculum species affecting electricity production. Liu and Li [10] suggested that the pure culture (*Rhodoferax ferrireducens*) and mixed cultures (activated sludge) possess similar electrochemical activity using monosodium glutamate wastewater (MGW) as the substrate; the effects of inoculums species on electricity production in a mediatorless MFC appear to be less significant.

### 3.3. Polarization Curves

**Figure 7:**
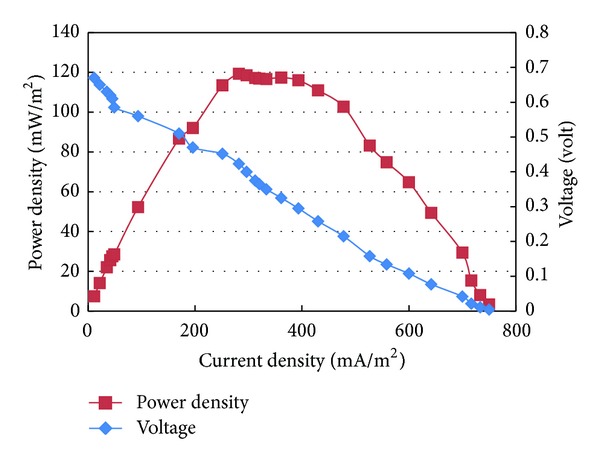
Polarization curve for MFC inoculated with activated sludge.

**Figure 8:**
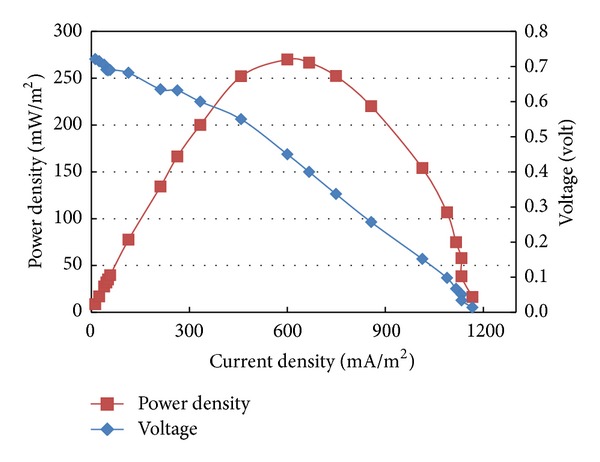
Polarization curve of MFC inoculated with single culture.

A polarization curve describes voltage as a function of current and is a powerful tool for the analysis and characterization of MFC. A maximum power can be produced when the internal and external resistances are equal [10]. In this study, for MFCs inoculated with mixed cultures and *Bacillus subtilis*, the relationships between the cell voltage and power densities as a function of the cell current densities are given in Figures 7 and 8, respectively. The plots present the power output from the MFC as a function of circuit load, using a periodical increase in the external variable resistor. A maximum power density of 120 mW/m^2^ at external resistance of 250 Ω was achieved in MFC inoculated with activated sludge (mixed cultures). The power density observed in this study is greater than a previously reported range of 2.1–70 mW/m^2^ [15–18] for MFC inoculated with activated sludge. The dissimilarity between the results obtained in this study and the previously reported studies inoculated with activated sludge could be attributed to the difference in abiotic parameters including but not limited to the quality of organic content concentration, type of wastewater, electrodes material, the applied resistance, and membrane type. On the other hand, for MFC inoculated with single culture (*Bacillus subtilis*), a maximum power density of 270 mW/m^2^ at an external resistance of 125 Ω was achieved. These results are significantly comparable to the power density of 300 mW/m^2^ obtained by Prakash et al. [19] using a polymeric-membrane MFC fed with lactate-based synthetic wastewater as the electron donor and inoculated with *Shewanella oneidensis* MR-1 as a single type of microorganism. Although, it is well known that *Shewanella oneidensis* MR-1 is an electrochemically active species, in this study *Bacillus subtilis* proved to be efficiently comparable to *Shewanella oneidensis* MR-1 taking into consideration the difference of substrate, type of membrane, and other abiotic factors.

However, for this study results revealed that MFCs inoculated with mixed and single cultures and fueled with actual domestic wastewater achieved different maximum power indicating the dissimilarity of electrochemical activity of the inoculum species. This could be attributed to the fact that the existence of electrochemically inactive species in the mixed cultures may compete with the active species for the available substrate and limit their activity and the subsequent released electrons.

### 3.4. Coulombic Efficiency

Coulombic efficiency deals with the electrons that are recovered from the substrate in the form of electric current. It expresses the rate of actual amount of electrons that is gained from the substrate in the form of electricity against the theoretical amount of electrons which are delivered by the bacteria based on the COD removal or substrate removal. The Coulombic efficiency is one of the most important indexes that were used to describe the MFC performance in terms of power generation. For continuous flow through the system, Coulombic efficiency can be calculated based on the generated current at steady-state conditions using (1) as cited by Logan et al. [2]:

(1) $$\begin{array}{c} \text{CE}=\frac{M\times I}{F\times b\times q\times \Delta S}\times 100\%, \end{array}$$

where *F* is the Faraday's constant (96485 Coulombs/mol-electron), *b* is the moles of electrons/mole of substrate, Δ*S* is the change in substrate concentration (g/L), *q* is the flow rate of substrate (L/sec), and *M* is the molecular weight of the substrate. The Coulombic efficiencies of the MFCs are given in Table 1.

However, in this study the results related to the columbic efficiency (CE) are higher or comparable to the previously reported values of CE. Liu et al. [20] recorded an increase in CE from 9.9 to 31.4% by decreasing the circuit resistance from 5000 to 70 Ω in a single-chambered MFC feed with 800 mg/L acetate solution. Fan et al. [21] reported a CE value of 35% using a single chamber air-cathode MFC fueled with 20–30 mM acetate solution. Futamata et al. [22] reported a 12.5% CE in a two-chamber MFC inoculated with anaerobic enrichment cultures with soil.

## 4. Conclusions

Simultaneous wastewater treatment and biological electricity generation were accomplished in the membrane MFC fueled with actual domestic wastewater as substrate and alternatively inoculated with activated sludge and *Bacillus subtilis.* Substrate degradation was clearly observed in the anode chamber of the microbial fuel cell in addition to renewable energy generation. Domestic electrogenic microbe *Bacillus subtilis* as a single pure culture was able to produce power and COD removal efficiency comparative to the mixed cultures in activated sludge. For MFC with single culture, its maximum power density was 270 mW/m^2^ while it was 120 mW/m^2^ for MFC with mixed cultures. Beside electricity generation, the MFCs were able to digest COD in wastewater, with 90 and 84% removal efficiencies using inoculums single and mixed culture, respectively.

## Figures and Tables

**Table 1:** Coulombic efficiency obtained from the MFCs.

MFC_(inoculum type)_	Maximum current (mA)	COD removal %	Δ*S* (mg/L)	CE %
MFC_(mixed culture)_	1.67	84	319	24.4
MFC_(single culture)_	3.60	90	338	49.6
